# The advantage of short paper titles

**DOI:** 10.1098/rsos.150266

**Published:** 2015-08-26

**Authors:** Adrian Letchford, Helen Susannah Moat, Tobias Preis

**Affiliations:** Data Science Lab, Behavioural Science, Warwick Business School, University of Warwick, Coventry CV4 7AL, UK

**Keywords:** citation analysis, scientific writing, computational social science, science of science

## Abstract

Vast numbers of scientific articles are published each year, some of which attract considerable attention, and some of which go almost unnoticed. Here, we investigate whether any of this variance can be explained by a simple metric of one aspect of the paper's presentation: the length of its title. Our analysis provides evidence that journals which publish papers with shorter titles receive more citations per paper. These results are consistent with the intriguing hypothesis that papers with shorter titles may be easier to understand, and hence attract more citations.

## 1. Introduction

Written communication is now being recorded online on a massive scale [1–5]. Colossal amounts of data on collective information gathering and distribution via online services such as *Twitter* [6–9], *Wikipedia* [10–13], *Google* [14–17], news services [18] and even large digitized collections of books [19–21] can now be analysed, widening our understanding of economic decision-making [11,14,16], human conflict [7,12] and natural disasters [22,23].

Scientific endeavours also generate extensive written communication, in the form of papers. We define a paper to be more successful than others if it has received a greater number of citations. The online database *Scopus* contains citation records of papers, offering remarkable insights into academic conversation. Recently, advances have been made in quantifying scientific output based on publication statistics [24–28]. A number of studies have provided evidence that the long-term success of scientists depends on their early publications [29,30]. Further analyses have indicated that a paper's success can be partially predicted by its early success [31–33] as well as the reputation of the authors [34]. In addition, papers in particular academic domains gain more citations than others [35].

Here, we consider whether we can find any evidence that the style in which a paper is written may relate to its success. Specifically, we consider the length of the article title chosen by the authors and investigate whether the length bears any relation to the number of citations. Previous studies have explored different characteristics of scientific paper titles [36–41]. A subset of these studies have focused on identifying stylistic attributes of academic writing and the use of a colon or question in a paper's title [36–39]. Those which have investigated the relationship between the length of an article's title and the number of citations it receives have been limited to relatively small samples, up to a maximum of 2200 papers [40,41]. These analyses have reported conflicting results, with one study suggesting that papers with longer titles might receive more citations [41] and another finding no evidence of a relationship [40]. Here, we exploit data on a much larger sample of 140 000 papers in order to investigate whether a paper's title length bears any relation to the number of citations it receives.

## 2. Results

We analyse data provided by *Scopus*, one of the leading bibliometric platforms. A *Scopus* user can search and export data on journal articles in batches of 20 000 records, including data on how often each article has been cited since publication. We download data on the 20 000 most cited papers in each year between 2007 and 2013.

**Figure 1.**
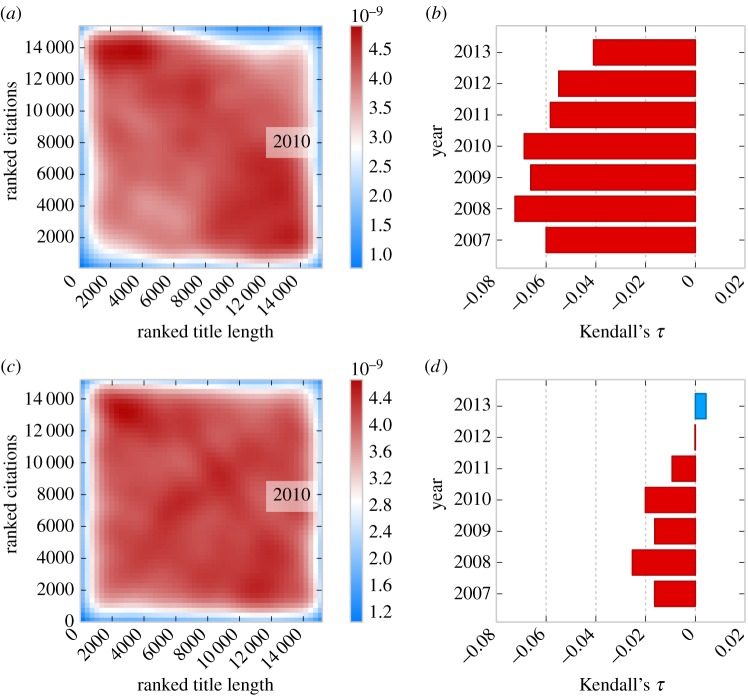
Paper title length and citations received. (*a*) We consider the 20 000 most cited papers in 2010. We rank the papers both in terms of citations received and title length. A density plot of the ranked citations and title length reveals that papers with shorter titles receive more citations (Kendall's *τ*=−0.07, *N*=15 395, *p*<0.001). (*b*) We run parallel analyses for the 20 000 most cited papers in each year between 2007 and 2013. For each of these years, we find that papers with shorter titles receive more citations (all *τ*s <−0.042, all *p*s <0.001, *α*=0.05, Kendall's *τ* correlation with FDR correction). (*c*) To remove any potential influence of the journal in which a paper is published on this relationship, we rank all of the papers in terms of the number of citations received and transform these ranks into percentiles. We calculate percentiles in terms of the length of papers' titles in the same fashion. In this transformed data, we find that papers with shorter titles receive more citations in 2010 (Kendall's *τ*=−0.020, *N*=15 395, *p*<0.001). (*d*) We run parallel analyses for the 20 000 most cited papers in each year between 2007 and 2013. For years 2007–2010, we find that papers with shorter titles receive more citations, whereas papers published during 2011–2013 do not (for years 2007–2010, all *τ*s <−0.016, all *N*s >14 791, all *p*s <0.01; for years 2011–2013, all |*τ*|s <0.01, all *N*s >15 396, all *p*s >0.05; Kendall's tau with FDR correction). The smaller *τ*s in (*d*) suggest that the journal in which a paper is published may help explain the relationship between paper title length and the number of citations the paper receives.

We determine the number of characters in each paper's title, including spaces and punctuation. Using the year 2010 as an example, we rank the papers' title length and citations (figure 1*a*). Upon visual inspection, there appears to be a high concentration of papers with short titles and many citations, as well as a high concentration of papers with long titles and few citations. We find that for the top 20 000 most highly cited papers published in 2010, papers with shorter titles receive more citations (Kendall's *τ*=−0.07, *N*=15 395, *p*<0.001). We apply the same analysis to each year in our sample and find that papers from all years exhibit this relationship between their title length and citations (figure 1*b*; all *τ*s <−0.042, all *p*s <0.001, *α*=0.05, Kendall's *τ* correlation with false discovery rate (FDR) correction).

Some journals may attract a greater number of citations for their papers owing to their reputation. To remove any potential influence of the journal in which a paper is published on the relationship between citations received and paper title length, we rank all of the papers in terms of the number of citations received and transform these ranks into percentiles. We calculate percentiles in terms of the length of papers' titles in the same fashion. In this transformed dataset, for papers published in 2010, we find that papers with shorter titles receive more citations (figure 1*c*; *τ*=−0.020, *N*=15 395, *p*<0.001, Kendall's *τ* correlation). Again, we run parallel analyses for the 20 000 most cited papers in each year between 2007 and 2013. For years 2007–2010, we find that papers with shorter titles receive more citations, whereas papers published during 2011–2013 do not (figure 1*d*; for years 2007–2010, all *τ*s <−0.016, all *N*s >14 791, all *p*s <0.01; for years 2011–2013, all |*τ*|s <0.01, all *N*s >15 396, all *p*s >0.05; Kendall's *τ* with FDR correction). These smaller *τ*s suggest that the journal in which a paper is published may help explain the relationship between paper title length and the number of citations the paper receives.

**Figure 2.**
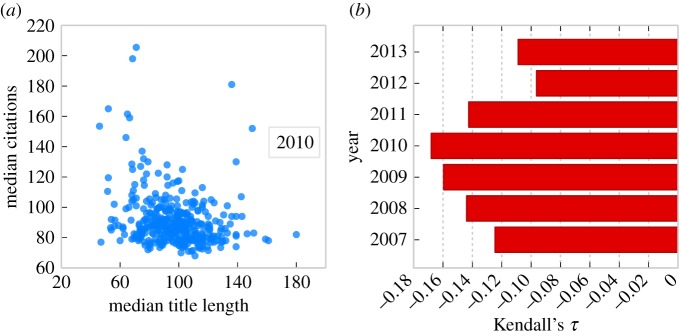
Paper title length and citations received, analysed at journal level. (*a*) For each journal in 2010, we plot the median citations for a paper against the median title length. We find that journals which publish papers with shorter titles receive more citations per paper (Kendall's *τ*=−0.19, *p*<0.001, *N*=361). (*b*) Parallel analyses of the data for each year between 2007 and 2013 confirm that this relationship holds across all 7 years of data (2012: *τ*=−0.1, *N*=320, *p*<0.05; 2013: *τ*=−0.11, *N*=352, *p*<0.01; all other years: all *τ*s≤−0.14, all *p*s <0.001, *α*=0.05; Kendall's *τ* correlation with FDR correction).

**Figure 3.**
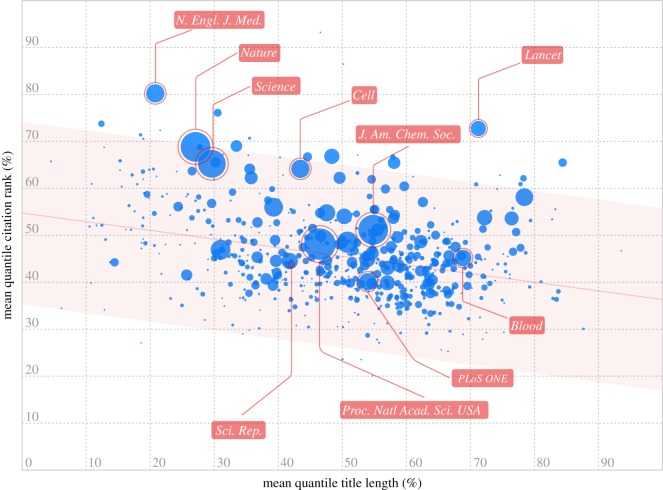
Journals which publish papers with shorter titles receive more citations per paper. For each year in our dataset, we rank all of the papers in terms of the number of citations received and in terms of the length of the titles, and transform these ranks into percentiles for a given year. For each journal, we then calculate the average quantile of the citations and of the title lengths, across papers and across years. Here, each blue circle represents a journal, the size of each circle represents the number of papers in our sample for that journal. Again, we find that journals that publish papers with shorter titles also tend to receive more citations per paper (Kendall's *τ*=−0.19, *N*=625, *p*<0.001).

To investigate this hypothesis further, we group papers by their journal. Again, using 2010 as an example, we calculate the median number of citations and median title length for each journal. We find that journals which published papers with shorter titles also tend to receive more citations per paper (figure 2*a*; Kendall's *τ*=−0.19, *N*=361, *p*<0.001). Parallel analyses for papers published in each year between 2007 and 2013 show that this relationship holds for papers published in all 7 years in our sample (figure 2*b*; 2012: *τ*=−0.1, *N*=320, *p*<0.05; 2013: *τ*=−0.11, *N*=352, *p*<0.01; all other years: all *τ*s≤−0.14, all *p*s <0.001, *α*=0.05; Kendall's *τ* correlation with FDR correction). Finally, we carry out a complementary aggregated analysis across all years of data in our sample. We rank all papers published in a given year by citations received and by title length, and transform these ranks into percentiles for that year. Again, we find that journals which publish papers with shorter titles also tend to receive more citations per paper (figure 3; *τ*=−0.19, *N*=625, *p*<0.001, Kendall's *τ* correlation).

Our primary analysis is based on rank-based statistics. To complement our analysis, we fit a mixed-effects model to the log of the number of citations a paper receives as a function of its title length controlling for the journal in which each paper is published. A mixed-effects models allows us to control for the journal in which each paper is published. We define our model as

2.1 $$\log_{10}(c_{j,p})=I+I_{j}+(L+L_{j})l_{j,p}+\epsilon_{j,p,}$$

where *c*_*j*,*p*_ is the number of citations received by paper *p* published in journal *j*. The distribution of citations received by a paper is highly positively skewed. For this reason, we log these citation counts, so that the distribution of the residuals of our model, *ϵ*, is closer to a Gaussian distribution. The grand intercept is *I*, whereas *I*_*j*_ is an intercept for each journal. There is a fixed slope *L* for the number of characters in the title *l*_*j*,*p*_ for paper *p* published in journal *j*. There is also a journal-level random effects slope for the title length *L*_*j*_. We fit the model for each year using maximum likelihood. We find that papers published during 2007–2011 with shorter titles tend to receive more citations while those published during 2012 and 2013 do not (for years 2007–2010: all *t*s <−3.832, all *p*s <0.001; 2011: *t*=−3.314, *N*=345 *p*<0.01; 2012–2013: both *t*s <−0.251, both *p*s >0.05; *t*-test on slope *L* with FDR correction). The values of the slope *L* are given for all years in table 1.

**Table 1.** Mixed effects model of the relationship between paper title length and citations received. Our primary analysis in figures 1 and 2 are based on rank statistics. To complement this analysis, we fit linear models to the data. We fit a mixed-effects model to the log of the number of citations a paper receives as a function of its title length (equation (2.1)). The model includes a fixed slope *L* for the number of characters in the length of a paper's title. We fit this model for each year in our dataset and display the slopes here under ‘for individual papers’. We find that, for each year, the slope is negative. We also investigate if this relationship exists when aggregating papers by the journal in which they are published. We fit a linear regression model to the log of the median number of citations papers receive per journal as a function of the median title length (equation (2.2)). There is a slope *L* for the median number of characters in the titles of papers published in each journal. We fit this model for each year in our dataset and display the slopes here under ‘for individual journals’. Again, we find that for each year, the slope is negative.

	slope of length (*L*)	
year	for individual papers	for individual journals
2007	−0.0118***	−0.0147***
2008	−0.0118***	−0.0208***
2009	−0.0093***	−0.0190***
2010	−0.0099***	−0.0174***
2011	−0.0078**	−0.0183***
2012	−0.0040	−0.0080*
2013	−0.0005	−0.0116**

Asterisks represent FDR-corrected *p*-values for a *t*-test of the slope. *p<0.05, **p<0.01, ***p<0.001.

Again, we investigate if this relationship exists when aggregating papers by the journal in which they are published. We fit a linear regression model to the median number of citations papers receive per journal as a function of the median title length. We define our model as

2.2 $$\log_{10}(c_{j})=I+Ll_{j}+\epsilon_{j},$$

where *c*_*j*_ is the median number of citations received by papers published in journal *j*. The intercept is *I*, and there is a slope *L* for the median number of characters in the titles of papers *l*_*j*_ published in journal *j*. Again, we log the citation counts, so that the distribution of the residuals of our model, *ϵ*, is closer to a Gaussian distribution. We fit the model for each year. We find that journals which publish papers with shorter titles also tend to receive more citations per paper (for years 2007–2011: all *t*s <−4.215, all *p*s <0.001; 2012–2013: both *t*s <−2.022, both *p*s <0.05; *t*-test of slope *L* with FDR correction). The values of the slope *L* are given for all years in table 1.

## 3. Discussion

In this study, we investigate whether the length of a scientific paper's title is related to the number of citations it receives. We analyse the 20 000 most highly cited papers for the years 2007–2013, representing a sample size between 1.12% and 1.53% of all papers published in each of these years. Previous studies analysing much smaller sets of papers have reported conflicting evidence, suggesting either that the length of a paper's title bears no relation to its scientific impact [40], or that longer titles can be linked to greater citation counts [41].

Our analysis suggests that papers with shorter titles do receive greater numbers of citations. However, it is well known that papers published in certain journals attract more citations than papers published in others. When citation counts are adjusted for the journal in which the paper is published, we find that the strength of the evidence for the relationship between title length and citations received is reduced. Our results do however reveal that journals which publish papers with shorter titles tend to receive more citations per paper.

We propose three possible explanations for these results. One potential explanation is that high-impact journals might restrict the length of their papers' titles. Similarly, incremental research might be published under longer titles in less prestigious journals. A third possible explanation is that shorter titles may be easier to understand, enabling wider readership and increasing the influence of a paper.

Our findings provide evidence that elements of the style in which a paper is written may relate to the number of times it is cited. Future analysis will investigate whether further stylistic attributes of the language used in a paper can be related to the number of citations it receives.

## 4. Methods

We retrieve bibliometric data from *Scopus* (http://www.scopus.com) between 21 October 2014 and 14 November 2014. To obtain data on the 20 000 most cited papers published in each of the 7 years from 2007 to 2013, we search for any papers that are marked by *Scopus* as an ‘article’ with the following search query:

DOCTYPE(ar) AND PUBYEAR = {year},

where {year} is replaced by each of the years 2007–2013. In total, we retrieve 140 000 records. In 2007, *Scopus* reports 1 302 973 published papers which increases to 1 788 065 papers in 2013. The top 20 000 most cited papers published in each year represent a sample of 1.53% in 2007, decreasing to 1.12% in 2013.

Some journals are referred to with multiple variations of their name (for example, ‘*Analyst*’ and ‘*The Analyst*’). For this reason, we clean the dataset from *Scopus* by deleting leading ‘The’s from each journal's title, and converting the title to lower case. We also identify all journals which have fewer than 10 papers in the most cited 20 000 papers for a given year, and remove the papers in such journals for that year. The basic characteristics of our dataset before and after cleaning are depicted in the electronic supplementary material, figure S1.

## Supplementary Material

Supplementary Information (Electronic document): Contains summary statistics of the dataset.

## Data accessibility

Datasets used in this study are available via the Dryad Repository (doi:10.5061/dryad.hg3j0).
