# Supplementary material for: The advantage of short paper titles
Source: R Soc Open Sci. 2015 Aug 26;2(8):150266. doi: 10.1098/rsos.150266 (PMC4555861; doi:10.1098/rsos.150266)
Supplement: Supplementary Information (Electronic document): Contains summary statistics of the dataset. [file rsos150266supp1.pdf]

The Advantage of Short Paper Titles

Supplementary Information

Adrian Letchford[1]*, Helen Susannah Moat[1,2,3], and Tobias Preis[1,2,3]

[1]Warwick Business School, University of Warwick, Coventry, CV4 7AL, UK

[2]Department of Physics, Boston University, 590 Commonwealth Avenue, Boston, Massachusetts 02215, USA

[3]Department of Mathematics, UCL, Gower Street, London, WC1E 6BT, UK

* To whom correspondence should be addressed; E-mail: Adrian.Letchford@wbs.ac.uk

July 24, 2015

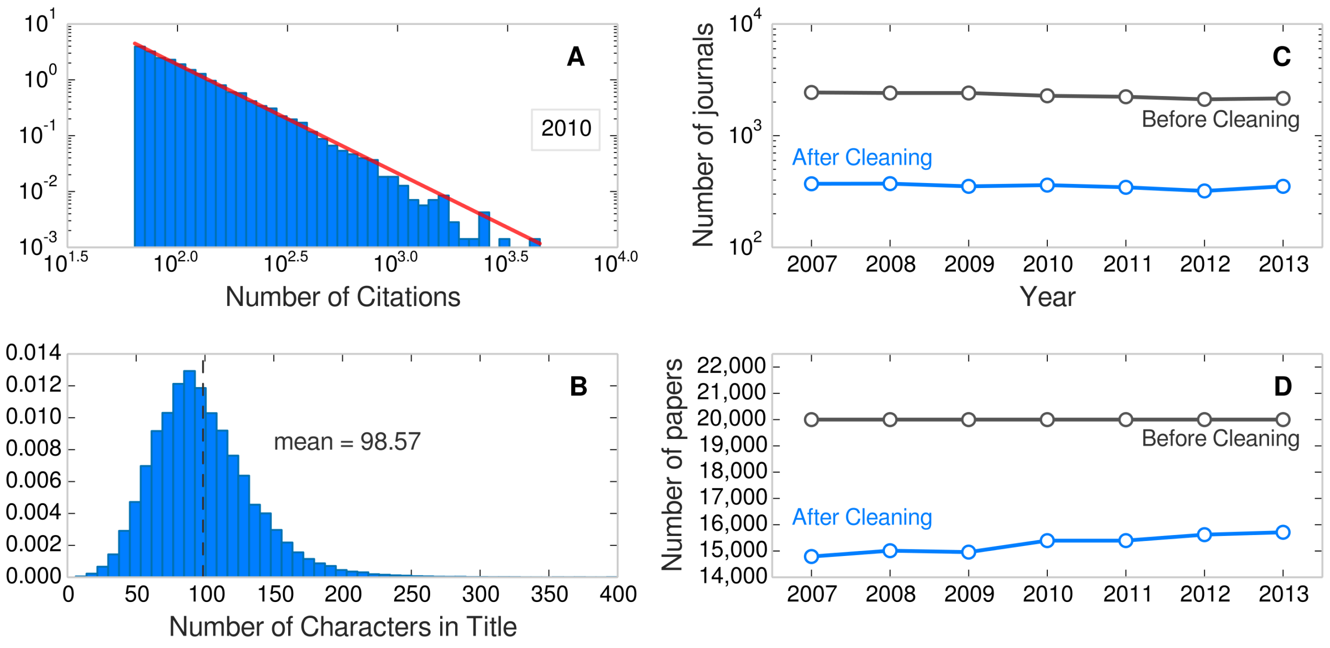

Figure S1: Basic properties of the citation dataset. (A) We analyse data on the 20,000 most cited papers published in each of the seven years from 2007 to 2013, where citation data was downloaded in Autumn 2014. Here, we depict the distribution of citations received by papers published in 2010, which we find is exponential. Parallel analyses reveal that the distributions of citations of papers published in the remaining six years are similarly exponential. **(B)** We analyse the lengths of the titles of papers in our sample. The mean length of a paper's title is 98.63 characters. **(C)** Each year, the most cited 20,000 papers are published in a mean total of 2,291 journals (grey line). To facilitate our core analysis, we identify all journals which have fewer than 10 papers in the most cited 20,000 papers for a given year, and remove the remaining papers in such journals for that year. A mean of 353.29 journals per year remain after this cleaning process (blue line). **(D)** Before cleaning, our dataset contains 20,000 papers per year (grey line). After cleaning, the mean number of papers per year is 15,269.57.
